# Correction: Gonçalves et al. Macrophage and Lymphocyte Infiltration Is Associated with Volumetric Tumor Size but Not with Volumetric Growth in the Tübingen Schwannoma Cohort. *Cancers* 2021, *13*, 466

**DOI:** 10.3390/cancers15020377

**Published:** 2023-01-06

**Authors:** Vítor Moura Gonçalves, Elisa-Maria Suhm, Vanessa Ries, Marco Skardelly, Ghazaleh Tabatabai, Marcos Tatagiba, Jens Schittenhelm, Felix Behling

**Affiliations:** 1 Department of Neurosurgery, University Hospital Tübingen, Eberhard-Karls-University Tübingen, 72076 Tübingen, Germany; 2 Faculty of Medicine, University of Porto, 4200-319 Porto, Portugal; 3 Center for Neuro-Oncology, Comprehensive Cancer Center Tübingen -Stuttgart, University Hospital Tübingen, Eberhard-Karls-University Tübingen, 72076 Tübingen, Germany; 4 Department of Neurology and Interdisciplinary Neuro-Oncology, University Hospital Tübingen, Eberhard-Karls-University Tübingen, 72076 Tübingen, Germany; 5 Hertie Institute for Clinical Brain Research, 72076 Tübingen, Germany; 6 German Cancer Consortium (DKTK), DKFZ Partner Site Tübingen, 72076 Tübingen, Germany; 7 Department of Neuropathology, Institute of Pathology and Neuropathology, University Hospital Tübingen, Eberhard-Karls-University Tübingen, 72076 Tübingen, Germany

## Text Correction

The authors would like to make a correction to their published paper [1]. We report that the formula for the calculation of the percentual volumetric tumor growth contained an error. The percentual growth was mistakenly calculated in regard to the preoperative tumor volume and not the first tumor volume that was measured as starting point of calculated tumor growth. We recalculated the percentual volumetric tumor growth for all included cases and provided updated CART-specific cut-offs. We now present the corrected results together with the updated figures and tables. The interpretation and discussion of the data are not affected by the recalculated values. In contrast to the original version of the article, an extreme outlier was excluded (one case with a volumetric tumor growth of 519.2%/year with the second fastest growth rate at 38.6%/year).

1. Paragraphs 2 and 3 in Section 2.2 should be corrected as:

Classification and regression tree analysis determined 1.57% MIB1 immunopositivity as the optimal cutoff in this cohort. Tumors with a MIB1 expression below 1.57% had a slightly larger preoperative tumor size compared to tumors with a higher MIB1 expression exceeding or equal 1.57%, but statistical significance was missed (4.93 compared to 4.03 cm^3^, *p* = 0.0770, see Figure 2A).

Significant differences in the mean preoperative tumor volume were also observed for all immune cell markers (CD3, CD8, CD68 and CD163). In contrast to MIB1, a higher preoperative tumor volume was seen for higher expression scores for all lymphocyte and macrophage markers, regarding the overall score. For the CART-derived cutoffs this was also the case except for CD3 (Figure 3 and Table 1).

2. Paragraphs 1 and 2 in Section 2.3 should be corrected as:

Volumetric tumor growth was assessed for 189 cases (20.5%). When regarding tumor growth as the difference in volume in cm^3^ per year, it showed similar significant differences across the inflammatory marker scores as the preoperative tumor volume. Thus, a higher expression score of CD3, CD8, CD68 and CD163 was associated with larger preoperative tumor size and faster volumetric growth in vestibular schwannomas (Figure S1). In contrast, the proliferative activity (MIB1 expression) did not show a significant difference in volumetric tumor growth in cm^3^ per year (Figure 2B). We next calculated the percentual volumetric tumor growth as a more representative marker for growth dynamic independent of the initial preoperative tumor volume. A MIB1 expression exceeding the cutoff at 1.57% had a significantly faster percentual volumetric tumor growth when compared to tumors with lower MIB1 expression (130.00 and 84.15%/year, respectively, *p* = 0.0032) (Figure 2C).

VS with a CD163 score of 0 showed a significant slower percentual tumor growth compared to tumors reaching a score of 1–4 (79.58 compared to 106.90%/year, respectively, *p* = 0.0465). Otherwise, the growth rate showed no difference when evaluating the immunohistochemical markers for lymphocyte (CD3 and CD8) and macrophage infiltration (CD68 and CD163). Results were not significant for the complete score and CART-specified cutoffs for each marker (Figure 4).

3. Paragraphs 1 and 2 in Section 2.4 should be corrected as:

For overall assessment an inflammatory score (IS) was generated from available immune cell marker data. An IS of 0 was given to 343 tumors (37.4%), meaning neither the CART-specified cut off for prominent lymphocytic nor macrophage infiltration was reached. A total of 535 cases (58.4%) received a score of 1 and 38 schwannomas (4.1%) reached the maximum score of 2, indicating prominent expression of lymphocyte and macrophage markers. 

The preoperative tumor volume was increased for tumors with a higher IS (Figure 5A). When both cohorts with scores 1 and 2 were combined the mean preoperative tumor volume reached 6.22 cm^3^, compared to 3.62 cm^3^ for tumors with a score of 0 (*p* < 0.0001, Figure 5B). In contrast to this, the percentual volumetric tumor growth was increased with higher inflammatory scores but without statistical significance (Figure 5C). 

4. Paragraph in Section 2.5 should be corrected as:

To assess the impact of the proliferative marker MIB1 and the inflammatory score, a multivariate linear regression was used, and the previously established CART-specified cut offs were applied (Table 2). A MIB1 expression exceeding 1.57% was revealed as an independent factor for faster tumor growth *p* = 0.0103). On the contrary, the inflammatory score did not reach statistical significance.

## Error in Figures and Tables

The new CART-specific cut-off for MIB1, according to the corrected calculated percentual volumetric growth rates, was 1.57% MIB1 immunopositivity. In contrast to the original version of the manuscript, differences in preoperative tumor volume according to MIB1 expression were not statistically significant. Percentual volumetric growth remained statistically significant (Figure 2).

The CART-derived cut-offs were recalculated based on the corrected percentual volumetric growth rates. Significant results for differences in preoperative tumor volumes according to CART-specific expression scores remained the same for all markers except for CD3 (Figure 3 and Table 1).

Now with the corrected values for percentual volumetric growth, vs. with a CD163 score of 0 showed a significantly slower percentual tumor growth compared to tumors reaching a score of 1–4 (79.58 compared to 106.90%/year, respectively, *p* = 0.0465). However, the overall score was without significant differences. All other markers remained without statistical significance (Figure 4).

Since the inflammatory score was based on the CART-specified results of the inflammatory markers, the score was recalculated as well. In the original version, we observed a slower percentual volumetric growth rate with a higher inflammatory score, which we were unable to explain properly. Now, the corrected growth rates provide an increase in volumetric growth with a higher inflammatory score but without statistical significance (Figure 5).

The multivariate analysis (linear regression) was recalculated with the corrected values. An expression of MIB1 above the CART-specific cut-off remained an independent factor for faster percentual volumetric tumor growth (*p* = 0.0103). In the original version of the manuscript, a higher inflammatory score was associated with slower tumor growth. Based on the corrected values, there is no statistically significant impact of the inflammatory score on percentual volumetric tumor growth (Table 2).

Furthermore, we would like to correct some minor transition mistakes in the first results, as in *Section 2.1 Distribution of Immunohistochemical Marker Expression*. A score of 0 for CD3 was seen in 162 and not 163 cases (17.6% instead of 17.7%). A score of 1 for CD3 was observed in 523/919 cases (56.9% instead of 57.6%). A score of 1 was reached for CD163 325/915 cases (35.5%) instead of 235/915 (25.7%).

The authors apologize for any inconvenience caused and state that the scientific conclusions are unaffected. The original article has been updated.

## Figures and Tables

**Figure 2:**
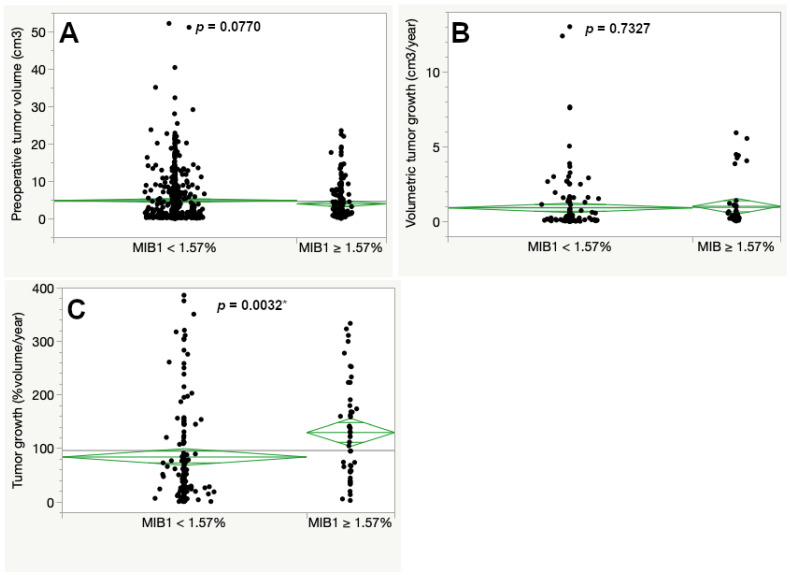
Preoperative tumor volume (**A**), volumetric tumor growth (**B**) and percentual volumetric (**C**). The asterisk (*) marks statistically significant results. Table 1 expression in the tumor tissue. The cutoff at 1.57% was set according to a classification and regression tree (CART) analysis regarding percentual volumetric growth (ANOVA, asterisk (*) marks statistically significant results).

**Figure 3:**
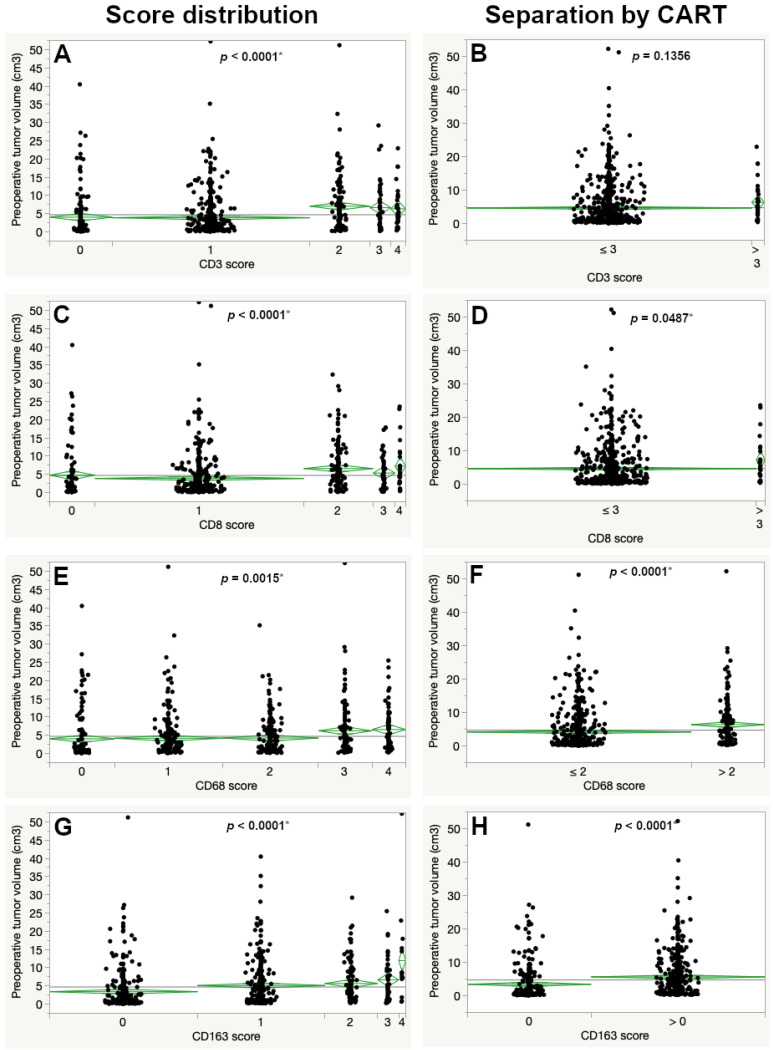
Differences in preoperative tumor volume according to the immunohistochemical expression across the complete immunohistochemistry score (left images) and after determining the CART-specific cutoff (right images) for CD3 (**A**,**B**), CD8 (**C**,**D**), CD68 (**E**,**F**) and CD163 (**G**,**H**). Significantly larger preoperative tumor volumes were seen with increased expression of each marker (ANOVA). The asterisk (*) marks statistically significant results.

**Figure 4:**
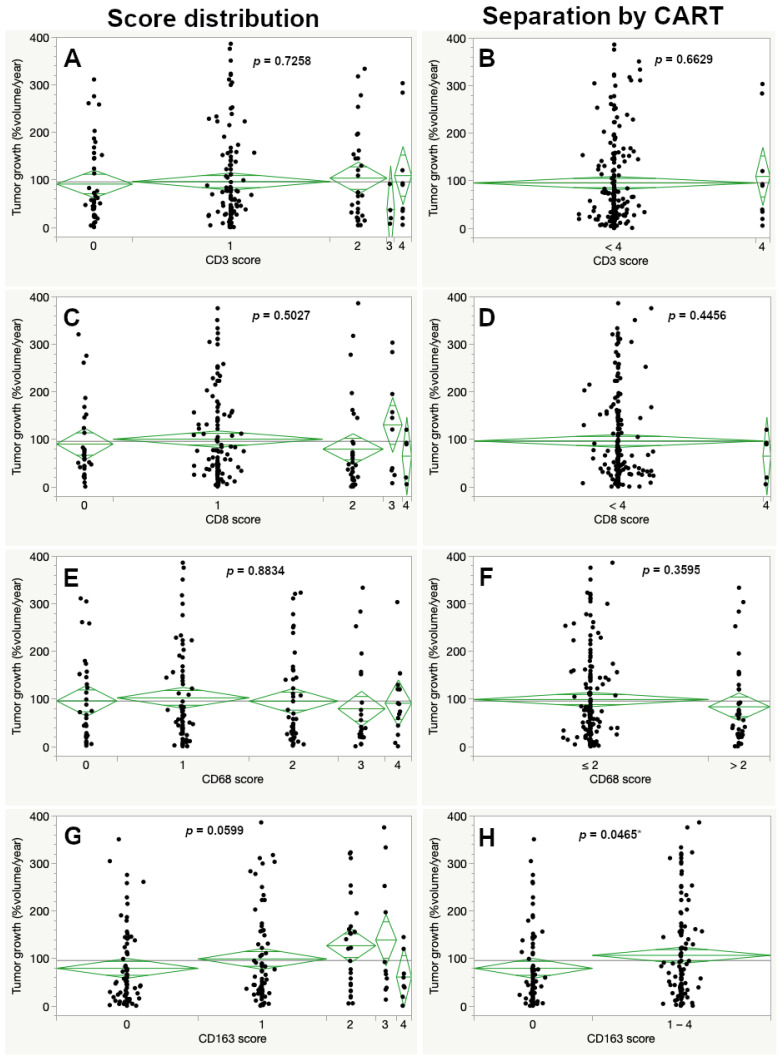
Percentual volumetric tumor growth according to the immunohistochemical expression for the complete immunohistochemistry score (left images) and the CART-specific cutoff (right images) for CD3 (**A**,**B**), CD8 (**C**,**D**), CD68 (**E**,**F**) and CD163 (**G**,**H**). No significant differences in tumor growth were seen, except for the CART-specific cut off for CD163 (ANOVA, asterisk (*) marks statistically significant results).

**Figure 5:**
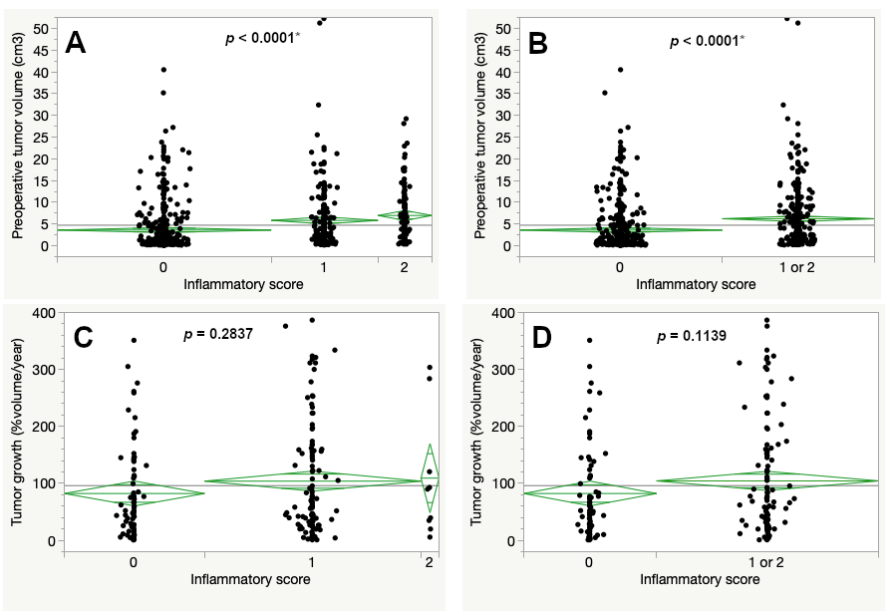
Preoperative tumor volume (**A**,**B**) and percentual volumetric tumor growth (**C**,**D**) according to the inflammatory score (ANOVA, asterisk (*) marks statistically significant results).

**Table 1:** Preoperative volumetry according to inflammatory cell marker expression.

Variable	*N* (%)	Mean Tumor Volume in cm^3^	*p*-Value (ANOVA)
CD3 score			
0	134 (17.5)	4.05	<0.0001 *
1	424 (55.5)	3.9	
2	129 (16.9)	7.06	
3	45 (5.9)	6.66	
4	32 (4.2)	6.34	
≤3	732 (95.8)	4.65	0.1356
>3	32 (4.2)	6.34	
CD8 score			
0	97 (12.7)	4.78	<0.0001 *
1	449 (58.7)	3.88	
2	149 (19.5)	6.6	
3	47 (6.1)	5.37	
4	23 (3.0)	7.24	
≤3	742 (97.0)	4.64	0.0487 *
>3	23 (3.0)	7.24	
CD68 score			
0	139 (18.2)	4.04	0.0015 *
1	231 (30.3)	4.19	
2	205 (26.9)	4.25	
3	115 (15.1)	6.23	
4	72 (9.5)	6.58	
≤2	575 (75.5)	4.18	<0.0001 *
>2	187 (24.5)	6.36	
CD163 score			
0	316 (41.5)	3.42	<0.0001 *
1	272 (35.7)	5.11	
2	113 (14.8)	5.67	
3	45 (6.0)	6.67	
4	15 (2.0)	11.97	
0	316 (41.5)	3.42	<0.0001 *
>0	445 (58.5)	5.64	
Inflammatory score			
0	435 (57.1)	3.62	<0.0001 *
1	217 (28.5)	5.84	
2	110 (14.4)	6.97	
0	435 (57.1)	3.62	<0.0001 *
1 or 2	327 (42.9)	6.22	

Asterisk (*) marks statistically significant results.

**Table 2:** Multivariate linear regression of percentual volumetric tumor growth.

	Estimate	Std Error	t Ratio	Lower 95%	Upper 95%	*p*-Value
Intercept	104.30	8.29	12.58	87.94	120.66	<0.0001 *
MIB1 ≥ 1.57%	−20.92	8.07	12.58	−36.83	−5.00	0.0103 *
Inflammatory score 0	−7.91	7.33	−1.08	−22.38	6.55	0.2818

Asterisk (*) marks statistically significant results.

## References

[1] Gonçalves V.M., Suhm E.-M., Ries V., Skardelly M., Tabatabai G., Tatagiba M., Schittenhelm J., Behling F. (2021). Macrophage and Lymphocyte Infiltration Is Associated with Volumetric Tumor Size but Not with Volumetric Growth in the Tübingen Schwannoma Cohort. Cancers.

