# Supplementary material for: Macrophage and Lymphocyte Infiltration Is Associated with Volumetric Tumor Size but Not with Volumetric Growth in the Tübingen Schwannoma Cohort
Source: Cancers (Basel). 2021 Jan 26;13(3):466. doi: 10.3390/cancers13030466 (PMC7865601; doi:10.3390/cancers13030466)
Supplement: Supplementary file 1 [file cancers-13-00466-s001.pdf]

cancers

MDPI

Supplementary Materials

Macrophage and Lymphocyte Infiltration Is Associated with Volumetric Tumor Size but Not with Volumetric Growth in the Tübingen Schwannoma Cohort

Vítor Moura Gonçalves, Elisa-Maria Suhm, Vanessa Ries, Marco Skardelly, Ghazaleh Tabatabai, Marcos Tatagiba, Jens Schittenhelm and Felix Behling

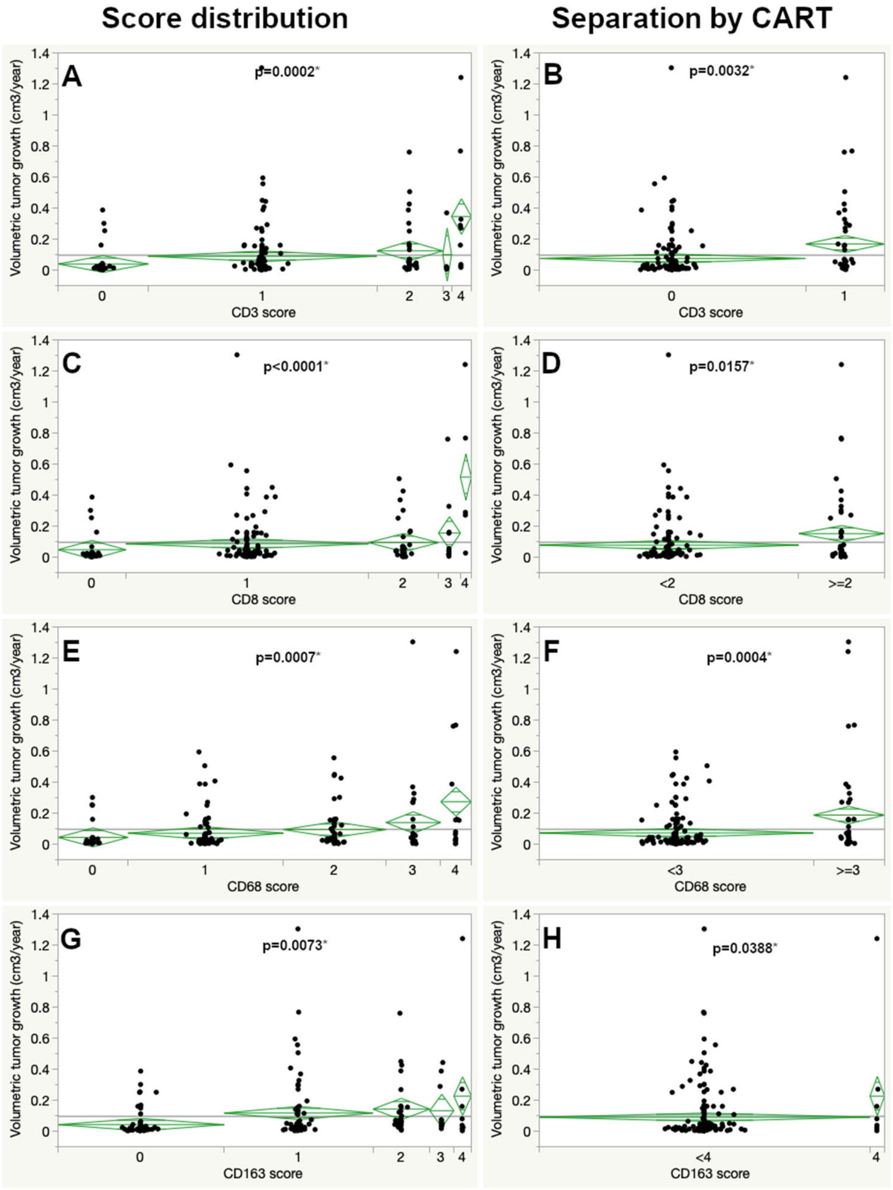

Figure S1. Volumetric tumor growth in cm^3/year according to the immunohistochemical expression for the complete immunohistochemistry score (left images) and the CART-specific cutoff (right images) Figure 3. (**A**,**B**), CD8 (**C**,**D**), CD68 (**E**,**F**) and CD163 (**G**,**H**). ANOVA, asterisk (*) marks statistically significant results.

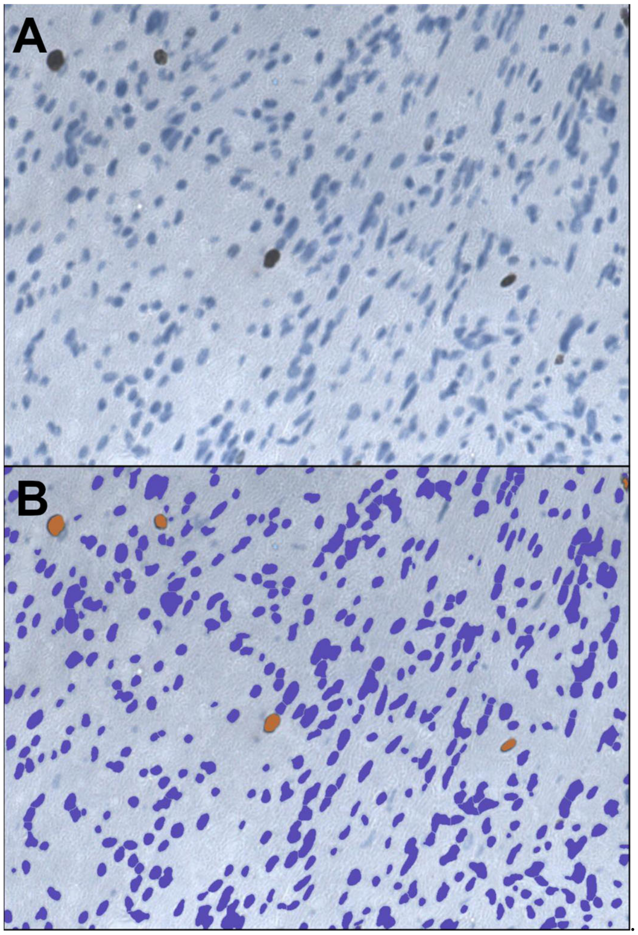

Figure S2. Immunohistochemical MIB1 expression and the automated digital expression quantification (magnification 200×).

Table S1. Characteristics of the study cohort.

**Variable**	***N* (%)**
Gender	
Female	484 (52.4)
Male	439 (47.6
MIB1	
<1.4%	573 (63.5)
>=1.4%	330 (36.5
CD3 score	
0	162 (17.6)
1	523 (56.9)
2	147 (16.0)
3	53 (5.8)
4	34 (3.7)
CD8 score	
0	118 (12.8)
1	544 (59.1)
2	176 (19.1)
3	55 (6.0)
4	27 (2.9)
CD68 score	
0	179 (19.5)
1	282 (30.8)
2	230 (25.1)
3	141 (15.4)
4	84 (9.2)
CD163 score	
0	386 (42.2)
1	325 (35.5)
2	134 (14.6)
3	54 (5.9)
4	16 (1.7)
Inflammatory score	
0	528 (57.6)
1	260 (28.4)
2	128 (14.0)
